# The more, the better? Stress response in related polyploid ferns 

**DOI:** 10.17912/micropub.biology.001591

**Published:** 2026-03-06

**Authors:** Steven Awad, Madeline Kenney, Jennifer Blake-Mahmud

**Affiliations:** 1 Biology Department, Hope College, Holland, Michigan, United States

## Abstract

Polyploidy, or whole genome duplication, is common in land plants. Polyploidy causes new gene combinations and is thought to provide an advantage amid the rising temperature and unpredictable precipitation regimes expected under climate change. Using related woodfern species, we investigated the gametophyte physiology of two parent species, *Dryopteris intermedia (2n)* and *Dryopteris expansa* *(2n)* in comparison to their polyploid offspring, *Dryopteris campyloptera (4n)* . We subjected gametophytes from each species to environmentally stressful heat and drought conditions. Surprisingly, the polyploid did not exhibit a significant physiological advantage or greater resilience to stress compared to the diploid parents.

**Figure 1. Measurements of gametophyte stress across time:**
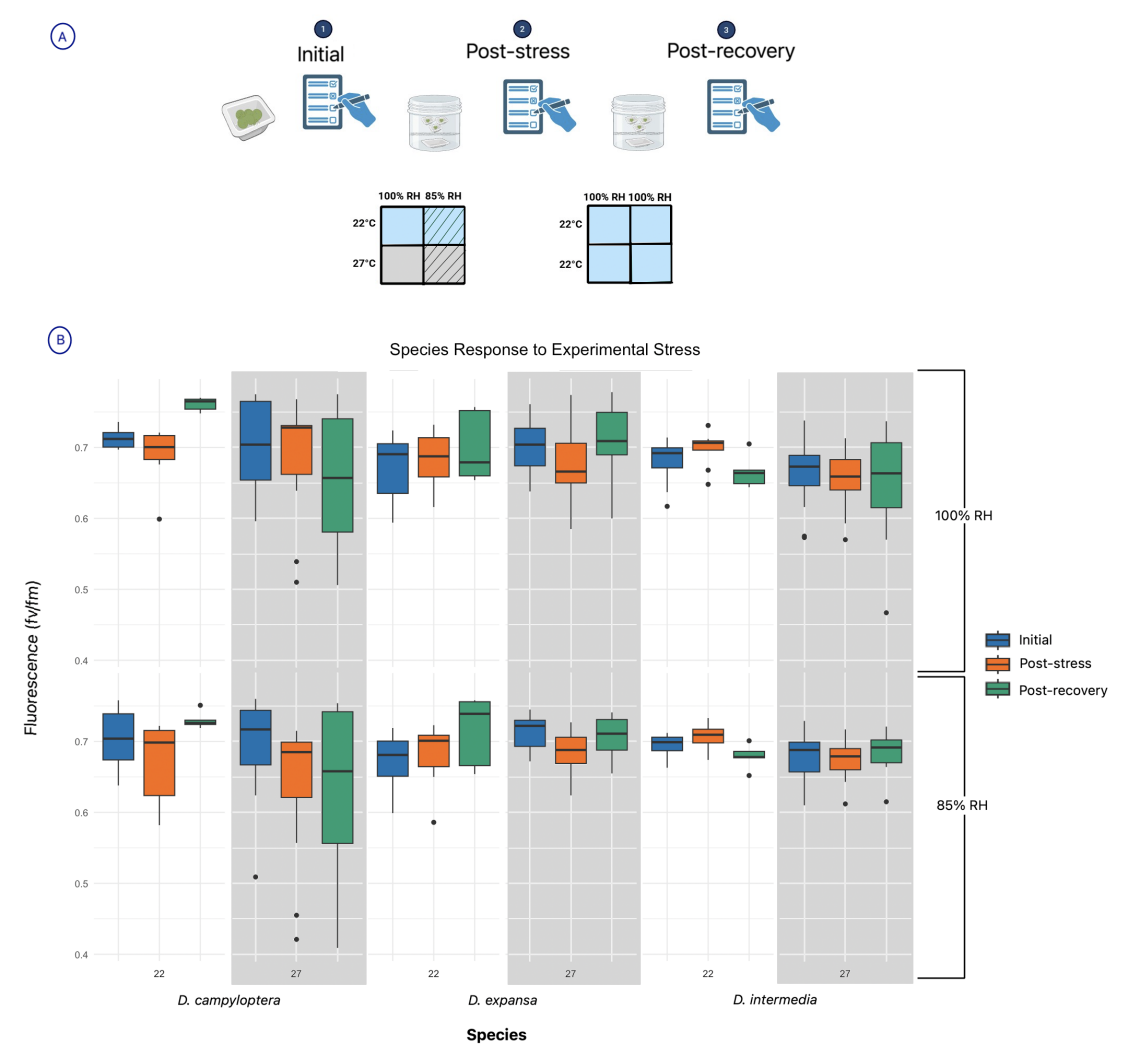
A. Experimental study design for stress response of diploids *Dryopteris expansa* and *Dryopteris intermedia* , and the tetraploid offspring, *Dryopteris campyloptera* . The schematic reflects our three recording measurement timepoints for fv/fm: (1) Initial pre-reading; (2) Post-stress reading; (3) Post-recovery reading. In between the first and second phases, the gametophytes underwent different treatments as indicated by the 2x2 table. The table displays experimental conditions such that relative humidity decreases from left to right and temperature increases from top to bottom, with control conditions in the upper left and the most stressful conditions in the bottom right. Following 48 hours in those treatments and another round of data collection, gametophytes returned to control conditions for 72 hours. B. Data collected during each measurement time point illustrate the response (post-stress) to and recovery (post-recovery) from stress. The top panel shows temperature stress at 100% RH; the bottom panel shows response at 85% RH. The colors blue, green, and orange represent time points. The background shading indicates an increased temperature condition of 27°C. The fluorescence values are on the y-axis, with lower values indicating more stress.

## Description

Polyploidy, or whole genome duplication, results in an individual possessing one or more extra sets of genetic material (Wood et al., 2009). Although polyploid species may incur difficulties associated with managing additional genetic material (Heslop-Harrison et al., 2022; Madlung, 2012), most studies herald the advantages brought about by polyploidy (Fox et al., 2020; Van de Peer et al., 2020), particularly under environmental stress conditions. Understanding the positive, negative, or neutral effect of polyploidy is critical, considering the essential role of allopolyploid woodfern species play within their mesic forest habitats (Wagner, 1971). Given the rising temperatures and extreme weather events associated with climate change (USGCRP, 2023), a more thorough understanding of the impact of stress on polyploids and related parent species is needed. As such, our study examines the effect of polyploidy on the physiological response to stress of the related parent ferns *Dryopteris intermedia* (2n) and *Dryopteris expansa* (2n), and their offspring *Dryopteris campyloptera* (4n).

Fern species are crucial to their habitats. They influence essential processes in their environments, including soil quality improvement (Lyu et al., 2019), and shading coverage for developing seedlings (Gould et al., 2013). Within the fern lifecycle, there are two distinct generations: the gametophyte and sporophyte (Krieg and Chambers, 2022). The haploid heart-shaped gametophyte is the fern’s sexual stage and is free-living (Krieg and Chambers, 2022). At this life-stage, the independent gametophyte is in equilibrium with its environment, lacking the homeostatic mechanisms of a mature sporophyte (Watkins et al., 2007b). It is largely dependent on its surrounding environment: if the environment is dry, the gametophyte is dry; if its surroundings are hot, the gametophyte is hot. After fertilization, the gametophyte produces a diploid zygote and enters its longest and dominant stage – the independent sporophyte generation in which the fern is self-sustaining and resistant to desiccation (Haufler et al., 2016).

We subjected the gametophytes of three related ferns, *Dryopteris intermedia* (2n), *Dryopteris expansa* (2n), and *Dryopteris campyloptera* (4n) to 48 hours of stress via drought (i.e., decreased relative humidity) and heat (i.e., increased temperature) experimental conditions. A linear mixed model (LMM) was built to evaluate the impact of the following predictors on stress levels: two 3-way interactions, Species*Time*Temperature, Species*Temperature*Relative humidity, and all nested lower-order interactions, with the individual as a random effect. We selected the final best-fitting model based on BIC. That final model contained a 3-way interaction between Species*Time*Temperature, all lower-order nested terms, and maintained the random effect of the individual. Surprisingly, the experimental parameter of relative humidity was not significant and worsened the model fit. Therefore, relative humidity was not included in the final model, thus indicating that this aspect of the experimental design did not constitute stress for these gametophytes. Model details are available in Table 1.

There was a statistically significant 3-way interaction between species, time, and temperature. This indicates that the difference in stress response across measurement timepoints varied based on temperature (p = 1.705e-05). This relationship was further impacted by species&nbsp; (p = 8.912e-05). We used emmeans to further investigate the interaction and found that the model-predicted response to stress between the initial and the post-stress timepoints was stronger within the 27-degree temperature treatment, particularly in the case of the tetraploid, *D. campyloptera.* More specifically, the model predicted a higher stress in 27-degrees for *D. campyloptera* (p = 0.0003). This points to the negative short-term effect of increased temperature on *D. campyloptera (* 4n). This evidence supports the idea that polyploidy does not always confer an immediate advantage in response to environmental stress.

These data are surprising, especially considering that an increase in genetic content might allow a synthesis of new genetically expressed characteristics that may help with combating stress (Leitch & Leitch, 2008; Ramsey, 2011). A study done on fern gametophytes in 2024 found that polyploidy enhanced the species’ ability to tolerate drought and heat stress conditions (Blake-Mahmud et al., 2024). Upon immediate comparison, these studies appear to contradict one another. However, it is crucial to acknowledge that the 2024 study saw the enhancing effects of polyploidy in more extreme conditions. The use of 75% relative humidity and 32℃ exposed the fern gametophytes to a greater amount of stress compared to the 85% relative humidity and 27℃ used in this study. The difference in response to varying severity of stress exposure led us to hypothesize that the polypoidal response to temperature and dryness may involve a threshold effect, beyond which gene activation occurs and mitigates the effects of the stress in the polyploid generation (Sade et al., 2012). As a result, the more moderate stress treatments in this experiment were insufficient to cross the threshold for subsequent increased response in gene activation. &nbsp;&nbsp;&nbsp;&nbsp;

Following the experimental stress, we placed the gametophytes in benign conditions (RH:100%, T: 22℃) to recover for 72 hours and measured their stress levels again. Despite *D. campyloptera’s* negative response to stress, a comparison between its initial and final fv/fm measurements indicates that there is no significant long-term effect of short-term temperature and drought stress (p = 0.2293). In fact, all three species were able to return to baseline following exposure to stress and subsequent time in cool, moist conditions (Table 1).&nbsp;

Much of the previous research done in this field has focused on stress response in autopolyploids, while our study assessed response in allopolyploids. The presence of a temperature-by-time-by-species interaction in our study suggests that while parent species might possess temperature-resistant genes of their own, those genes might not continue to have the same additive effect when introduced into a hybrid genome in a polyploid offspring. It has been suggested that long periods of consistent exposure to selective factors, particularly to stress, might be needed to hone expression select for resiliency in polyploid genomes (Maherali et al., 2009; Ramsey, 2011). While other research points to the physiological benefit that polyploids appear to hold in the face of environmental stress (Sessa 2018, Van de Peer et al., 2020; Heslop-Harrison et al., 2022), our study indicates no immediate benefit to polyploidy under stressful conditions when compared to their parent diploids. These results could indicate that whole genome duplication alone does not generate an advantageous response (Maherali et al., 2009) to all levels of stress, indicating that the physiological effect of polyploidy on stress may not be linear. This non-linear relationship would suggest that there is a threshold of stress at which the effect of polyploidy on stress response is further amplified and observed (Sade et al., 2012). While our study used mild stress conditions across a short period of time, higher stress levels and longer exposure might reveal more beneficial effects of polyploidy.&nbsp;

Table 1. This table presents the ANOVA results of the best-fit model and the corresponding statistical data.

**Table:** 

Variable	DF	F-value	P-value
Species	2, 144.68	1.515	0.223
Temperature	1, 144.68	4.648	0.0328
Time	2, 222.98	3.950	1.958e-06
Species: temperature	2, 144.68	2.268	0.107
Species: time	4, 222.98	8.419	2.420e-06
Temperature: time	2, 222.98	1.5381	1.705e-05
Species: temperature: time	4, 222.98	6.2396	8.912e-05

Table 2. This table depicts the estimated marginal means for the best-fit model. Pairwise comparisons are displayed between timepoints at a given temperature by Species. Statistically significant results are in bold.

**Table:** 

	22		27	
Species	p-value	estimate	p-value	estimate
Initial to Post-Stress				
*D. campyloptera*	0.1899	0.027	**0.0003**	**0.037**
*D. intermedia*	0.9419	—	1.00	—
*D. expansa*	0.9994	—	0.1064	0.0255
Post-recovery to Post-Stress				
*D. campyloptera*	<0.0001	0.0699	0.9998	—
*D. intermedia*	0.9164	—	0.9995	—
*D. expansa*	0.1075	0.03795	0.7941	—
Initial to Post-Recovery				
*D. campyloptera*	0.03	-0.0428	**0.2293**	**0.0277**
*D. intermedia*	1.00	—	1.00	—
*D. expansa*	0.0053	-0.0485	1.00	—

## Methods

Species&nbsp;

The Dryopteris genus includes many polyploids and is distributed throughout North America (Sessa et al., 2012). We collected frond tissue during sporulation from fern sporophytes growing in the understories of mesic forested regions in the following three locations: *D. expansa* from Kincaid Park in Anchorage Alaska (61.154, -150.062), *&nbsp;D. campyloptera* from Smugglers Notch in Cambridge Vermont (44.543, -72.787), and *D. intermedia* from North Point Nature Preserve in Charlevoix Michigan (45.338, -85.248). *Dryopteris expansa* and *Dryopteris campyloptera* were found in monotypic stands, while *Dryopteris intermedia* grew with other fern genera.&nbsp;

Treatment

Spores from all three groups of species were sown on agar plates. Once they grew into mature gametophytes under low light levels (10-20 PAR), we took a clump of gametophytes 1 cm in diameter and placed them in weigh boats with moist soil. To understand initial fern physiological health, we measured the fv/fm using a PAM-2500 Chlorophyll Fluorometer (Walz Photosynthesis Instruments, Effeltrich, Germany). Used as a standard plant health indicator, fv/fm is a ratio of the minimum and maximum fluorescence emitted by the photosystem II (PSII) of plants, enabling the assessment of light absorption ability and efficiency by plants (Mohammed et al., 2003; Murchie and Lawson, 2013; Wu et al., 2023). This measurement reflects the plant’s photosynthetic health and resilience, with higher fv/fm scores indicating low stress and high efficiency, and lower fv/fm scores indicating high stress and low efficiency.

Weigh boats were then separated into different containers, each containing different relative humidity conditions simulated using the saturated salt method (Watkins et al., 2007a; Rockland, 1960): (1) 100% relative humidity (i.e., high relative humidity) or (2) 85% relative humidity (i.e., low relative humidity; Ψ = -22.2 MPa for 22 degrees; Ψ = -22.5&nbsp; for 27 degrees). We then placed the species into the growth chamber (Conviron GEN1000) at either 22° C (i.e., cool temperature) or 27° C (i.e., warm temperature). The conditions reflected the four following tests: (1) 100% relative humidity at 22° C (n=30); (2) 100% relative humidity at 27° C (n=39); (3) 85% relative humidity at 22° C (n=30); (4) 85% relative humidity at 27° C (n=39). All of these were kept in their respective treatments in the dark for two days, with temperatures dropping 5° C for 12 hours each night. *In situ* , gametophytes often grow under dense leaf litter, which impedes light penetration. Light can be an additional stressor, so gametophytes were kept in the dark, following previously published experimental protocols (Chambers et al., 2017). After 48 hours, we removed the species from the growth chamber and took fluorescence measurements to obtain their post-treatment fv/fm value. Groups undergoing a recovery phase were placed into the growth chamber for an additional 72 hours in the control treatment condition – 100% relative humidity and 22° C. After 72 hours of recovery, we took their fv/fm values in order to compare health from before and after recovery (n= 69).&nbsp;&nbsp;

Data Analysis Software

The statistical analysis and visualization performed in this experiment were done using the R Project programming software (R version 4.4.0; RStudio version 2024.04.1+748; R Core Team, 2025). The packages used in R included: lme4 (Bates et al., 2015), emmeans (Lenth et al., 2025), dplyr (Wickham et al., 2025), and ggplot2 (Wickham, 2016).

We built a linear mixed model to evaluate the impact of the experimental treatment on stress levels (fv/fm). Predictors included two 3-way interactions, Species*Time*Temperature, Species*Temperature*Relative humidity, and all nested lower-order interactions, with a random effect for individual to account for repeated measures. Model selection was based on the lowest BIC, with non-significant terms removed until BIC worsened. Relative humidity was not significant and worsened the model fit, therefore it was removed from the model. The final model contained a 3-way interaction between Temperature, all lower-order nested terms, and maintained the random effect of the individual.

The graphs were created on R using the package ggplot2. Figure 1a was created through the online design tool Canva.com.

## References

[R1] Bates Douglas, Mächler Martin, Bolker Ben, Walker Steve (2015). Fitting Linear Mixed-Effects Models Using
**lme4**. Journal of Statistical Software.

[R2] Blake‐Mahmud Jennifer, Sessa Emily B., Visger Clayton J., Watkins James E. (2024). Polyploidy and environmental stress response: a comparative study of fern gametophytes. New Phytologist.

[R3] Chambers Sally M., Watkins J. E., Sessa Emily B. (2017). Differences in dehydration tolerance among populations of a gametophyte‐only fern. American Journal of Botany.

[R4] Fox Donald T., Soltis Douglas E., Soltis Pamela S., Ashman Tia-Lynn, Van de Peer Yves (2020). Polyploidy: A Biological Force From Cells to Ecosystems. Trends in Cell Biology.

[R5] Gould Rachelle, Mooney Harold, Nelson Laura, Shallenberger Robert, Daily Gretchen (2013). Restoring Native Forest Understory: The Influence of Ferns and Light in a Hawaiian Experiment. Sustainability.

[R6] Haufler Christopher H., Pryer Kathleen M., Schuettpelz Eric, Sessa Emily B., Farrar Donald R., Moran Robbin, Schneller J. Jakob, Watkins James E., Windham Michael D. (2016). Sex and the Single Gametophyte: Revising the Homosporous Vascular Plant Life Cycle in Light of Contemporary Research. BioScience.

[R7] Heslop-Harrison J S (Pat), Schwarzacher Trude, Liu Qing (2022). Polyploidy: its consequences and enabling role in plant diversification and evolution. Annals of Botany.

[R8] Krieg Christopher P., Chambers Sally M. (2022). The ecology and physiology of fern gametophytes: A methodological synthesis. Applications in Plant Sciences.

[R9] Lenth R, Piaskowski J (2025). *emmeans: Estimated Marginal Means, aka Least-Squares Means* . R package version 2.0.1, https://rvlenth.github.io/emmeans/.

[R10] Leitch AR, Leitch IJ (2008). Genomic plasticity and the diversity of polyploid plants.. Science.

[R11] Lyu Maokui, Xie Jinsheng, Giardina Christian P., Vadeboncoeur Matthew A., Feng Xiaojuan, Wang Minhuang, Ukonmaanaho Liisa, Lin Teng-Chiu, Kuzyakov Yakov, Yang Yusheng (2019). Understory ferns alter soil carbon chemistry and increase carbon storage during reforestation with native pine on previously degraded sites. Soil Biology and Biochemistry.

[R12] Madlung A (2012). Polyploidy and its effect on evolutionary success: old questions revisited with new tools. Heredity.

[R13] Maherali Hafiz, Walden Alison E., Husband Brian C. (2009). Genome duplication and the evolution of physiological responses to water stress. New Phytologist.

[R14] Mohammed Gina H., Zarco-Tejada Pablo, Miller John R. (2003). Applications of Chlorophyll Fluorescence in Forestry and Ecophysiology. Practical Applications of Chlorophyll Fluorescence in Plant Biology.

[R15] Murchie E.H., Lawson T. (2013). Chlorophyll fluorescence analysis: a guide to good practice and understanding some new applications. Journal of Experimental Botany.

[R16] R Core Team. 2025. R: A language and environment for statistical computing.

[R17] Ramsey Justin (2011). Polyploidy and ecological adaptation in wild yarrow. Proceedings of the National Academy of Sciences.

[R18] Rockland L. B. (1960). Saturated Salt Solutions for Static Control of Relative Humidity between 5° and 40° C.. Analytical Chemistry.

[R19] Sade Nir, Gebremedhin Alem, Moshelion Menachem (2012). Risk-taking plants. Plant Signaling & Behavior.

[R20] Sessa Emily B. (2018). Polyploidy as a mechanism for surviving global change. New Phytologist.

[R21] Sessa Emily B., Zimmer Elizabeth A., Givnish Thomas J. (2012). Phylogeny, divergence times, and historical biogeography of New World
*Dryopteris*
(Dryopteridaceae). American Journal of Botany.

[R22] USGCRP (2023). Fifth National Climate Assessment.

[R23] Van de Peer Yves, Ashman Tia-Lynn, Soltis Pamela S, Soltis Douglas E (2020). Polyploidy: an evolutionary and ecological force in stressful times. The Plant Cell.

[R24] Wagner, W. H. (1971). Ferns in Biology: Some Final Comments. BioScience.

[R25] Watkins James E., Mack Michelle C., Sinclair Thomas R., Mulkey Stephen S. (2007). Ecological and evolutionary consequences of desiccation tolerance in tropical fern gametophytes. New Phytologist.

[R26] Watkins James E., Mack Michelle K., Mulkey Stephen S. (2007). Gametophyte ecology and demography of epiphytic and terrestrial tropical ferns. American Journal of Botany.

[R27] Wickham Hadley (2016). ggplot2. Use R!.

[R28] Wickham Hadley, François Romain, Henry Lionel, Müller Kirill, Vaughan Davis (2014). dplyr: A Grammar of Data Manipulation. CRAN: Contributed Packages.

[R29] Wood Troy E., Takebayashi Naoki, Barker Michael S., Mayrose Itay, Greenspoon Philip B., Rieseberg Loren H. (2009). The frequency of polyploid speciation in vascular plants. Proceedings of the National Academy of Sciences.

[R30] Wu Qiang, Zhang Yongping, Xie Min, Zhao Zhiwei, Yang Lei, Liu Jie, Hou Dingyi (2023). Estimation of Fv/Fm in Spring Wheat Using UAV-Based Multispectral and RGB Imagery with Multiple Machine Learning Methods. Agronomy.

